# Water Carcinogenicity and Prevalence of HPV Infection in Esophageal Cancer Patients in Huaihe River Basin, China

**DOI:** 10.1155/2018/2028986

**Published:** 2018-05-06

**Authors:** Maliha Ghaffar, Jintao Li, Lei Zhang, Sara Khodahemmati, Minglian Wang, Yangjunqi Wang, Lijiao Zhao, Runqing Jia, Su Chen, Yi Zeng

**Affiliations:** ^1^Beijing Key Laboratory of Environmental and Viral Oncology, College of Life Science and Bio-Engineering, Beijing University of Technology, Beijing, China; ^2^China National Center for Food Safety Risk Assessment, Beijing, China; ^3^Hubei Key Laboratory of Medical Information Analysis & Tumor Diagnosis and Treatment, Hubei, China; ^4^National Institute for Viral Disease Control and Prevention, Chinese Center for Disease Control and Prevention, and State Key Laboratory for Infectious Disease Prevention and Control, Beijing, China

## Abstract

**Objective:**

The incidence of the upper gastrointestinal tumor has increased rapidly during recent decades. The relationship between local water pollution and the tumor is still not much clear, so this study was conducted to further investigate the local water pollution and its influence on the malignant cell transformation. Prevalence of human papillomavirus (HPV) in local esophageal cancer (EC) patients was also analyzed in Shenqiu County for the first time.

**Methods:**

Two-step cell transformation was used to study different sources of water in the malignant cell transformation, and the existence of 3-methylcholanthrene (3-MC) in water was analyzed from the river and shallow and deep wells. HPV DNA in tissue samples of EC patients was detected by polymerase chain reaction (PCR) and HPV diagnostic kit.

**Results:**

The river water has higher cytotoxicity than the shallow well water and induced significant cell malignant transformation, while deep well water has not shown the malignant cell transformation. In Huaihe River water, the 3-MC concentration was found higher than shallow and deep wells. An HPV infection rate was found high in patients with esophageal cancer.

**Conclusion:**

Long-term consumption of polluted water can induce malignant cell transformation, and the presence of HPV may be an important cause of cancer.

## 1. Introduction

In recent years, the rapid economic growth in China has amplified the polluted water in several main rivers and lakes, resulting in substantial threats to the health of people living adjacent to them [1]. Since the 1990s, there is the rapid development of agricultural and industrial production to fulfill the needs because there is a dramatic increase in population. Along with this, the increase of township enterprises in the Huaihe River Basin has led to an ever-increasing amount of wastewater from industries and factories; rubbish from urban areas, domestic sewage, medical waste, and pesticides and fertilizers from farmland are discharged into the river [2–8]. This rapid development in industrialization degraded the environment and caused deterioration of water quality [9].

Since 2000, the health of people has been badly affected due to change in the environment. Upper gastrointestinal malignant tumors including esophageal cancer are the important reason for the morbidity and mortality of cancer, especially in the developing countries [8]. “Cancer village” is a common term used for a village in which the rate of cancer morbidity is significantly higher as compared to the average level, and environmental pollution is considered as the key reason for it [10]. In China, Huaihe River Basin and mid-lower Yellow River Basin are the major locations of these cancers [11]. About 70% of surface water is not found suitable for drinking in China [12]. Treated water is available for drinking in urban areas while untreated drinking water is used in rural areas; therefore, the cancer morbidity is higher in rural areas as compared to urban areas. China has faced an increase in rural cancer rates in the 1990s [1, 9]. There is a correlation found between quality of water and digestive tract mortality as well as with distance of residential areas from polluted water bodies like rivers and lakes [1, 13]. 81% of the cancer villages are located in areas having less than 5 km distance from the major polluted river. Morbidity and mortality of digestive tract cancers are significantly higher in the areas closer to polluted rivers than distant areas [1, 14]. Water quality grades are found to have a more strong correlation with cancer mortality as compared to the distance from polluted water bodies [9, 13–15].

Gastric cancer and esophageal cancer (EC) are the most common upper gastrointestinal tumors in China; both are present with obvious regional distribution and high incidence of kiln distribution in the rural and impoverished mountainous area [16]. There is a rapid increase of Huaihe River Basin research, and upper gastrointestinal tumor cancer research has also been increased accordingly. The pathogenesis of cancer is not clear because cancer is a multistage and multifactor complex process. Research into the causes of upper gastrointestinal tract tumor related to environmental pollution is reported in many studies. Most of the studies investigated persistent organic pollutants (POPs) present in water. The contamination of the water has been seriously affecting the living standard of the people residing along the rivers having polluted water [11, 17, 18].

Shenqiu County is located in the Henan province. The county covers a total area of 1080.53 square kilometers. The incidence of gastric cancer and esophageal cancer has risen as compared to the national level and mortality rate because esophageal cancer is about 119.71% higher than the national level. At present, the rate of cancer death is increasing and the rapid increase of cancer mortality has become a key concern. Shenqiu County area is indeed an area to be further studied because of an increased death rate due to cancer while there is a decline in mortality due to cancer in other regions of China. It is documented that each year, approximately 2000 residents of this area died because of cancer out of 1,293,100 population. Water pollution is linked to cancer in different studies [6, 19].

The association of HPV with EC was first hypothesized in 1982 [20]. The relationship of HPV with EC is studied by many researchers, and HPV is now considered as the risk factor for EC. HPV16 was also found integrated into human chromosomes. The relationship of HPV high-risk types with gastrointestinal cancers is documented in recent studies from Iran and China [21, 22].

There is no report on the detection of carcinogenicity in the Huaihe River Basin from Shenqiu County; therefore, this study is conducted to explore in depth the local water source for malignant cell transformation, indirect evaluation of tumor, local tumor-causing HPV analysis, and evaluation of 3-MC. The objective of the research was to investigate the combined action of carcinogens and HPV in causing EC. The study of this kind was important in order to provide important clues about the relationship of local EC with HPV and pollution.

## 2. Materials and Methods

### 2.1. Cell Preparations

Balb/c 3T3 cells, clone ATCC CCL-163, were maintained in our laboratory. Balb/c 3T3 cell line was used for transformation assays due to its sensitivity to exogenous carcinogens. Cells were cultivated in a Dulbecco's modified Eagle medium with high glucose (HyClone, Beijing, China) supplemented with 10% fetal bovine serum and antibiotics (penicillin and streptomycin, 100 U/mL) under standard conditions (37°C, 5% CO_2_). Exponentially proliferating cells (2 × 10^6^) were washed with phosphate-buffered saline (PBS) of pH 7.4 (HyClone, Beijing, China) and harvested by centrifugation (Thermo Scientific CL31, USA) at 1000 rpm for 5 minutes after digestion with 0.25% trypsin (HyClone, Logan, USA).

### 2.2. Detection of HPV DNA

Tissue samples of esophageal cancer patients were collected randomly from Shenqiu People's Hospital. A total of 66 samples were collected, and all were included for detection of HPV DNA. For this purpose, genomic DNA was isolated from cells according to the manufacturer's protocol by using the QIAamp DNA mini kit (Qiagen, Germany). HPV DNA of the esophageal cancer specimens in the Huaihe River Basin was detected by polymerase chain reaction (PCR) by using general primer sets of My09/11 and GP5+/6+ for conserved genes of HPV. The type-specific primer sets were designed according to the HPV16 and 18 gene sequences in GenBank. The type-specific HPV were detected according to the protocol of Kaipu-type HPV detection kit (Kaipu, Chaozhou, China). The general primer sets for HPV DNA detection are listed in Table 1. PCR was conducted according to Jacobs. The PCR products were examined by gel electrophoresis with 1.2% agarose.

### 2.3. Water Collection and Pretreatment

Water samples were collected from Shenqiu County of Henan province from the Dong Cai He section of the river and from the Shenqiu County shallow well and deep well for cancer rate. Oasis® HLB solid-phase extraction column (1 g, Waters Company, USA) is used for the enrichment of water samples. The elution process was as follows: (1) balance/activation, 5 mL of methanol in turn and 5 mL deionized water; (2) the enrichment of sample, sample amount 3 L in each column; (3) washing column, 5% methanol 10 mL of water; (4) elution/collection, methanol 10 mL; (5) concentrate preparation, rotary evaporation to about 1 mL and nitrogen blow to nearly dry; and (6) constant volume with DMSO. Sample processing information is shown in Table 2. For cell transformation experiment, the concentrate was diluted in proportion to the cell culture medium.

### 2.4. Cell Transformation Assay

Balb/c 3T3 cells were seeded at a density of 1 × 10^4^ cells/25 cm^2^ flask and treated with Ad-18 for 3 days. On the 4th day, the treatment medium was replaced with DMEM/F12 (1 : 1) medium, supplemented with 0.2% ITES (1.0 mg/mL recombinant human insulin, 0.55 mg/mL human transferrin, 0.5 *μ*g/mL sodium selenite, and 0.2 mg/mL ethanolamine, from Sigma-Aldrich) and 2% FBS. The cultures were maintained for 3 days. Then, the cultures were treated with 0.1 *μ*g/mL 12-*O*-tetradecanoylphorbol-13-acetate (TPA) as the promoter for one week. When TPA exposure was terminated, the cultures were maintained in the DMEM/F12 (1 : 1) medium, which was changed twice a week until transformation. Typically, the assay duration was 5-6 weeks.

After cultivation for 6 weeks, the cells were seeded in a 6-well cell culture plate, prepared with ultrapure water with two concentrations of agarose solution, respectively, 1.2% and 0.7%. Then, they were placed in a water bath at 37°C after high-pressure sterilization. In 2x DMEM medium, 20% fetal bovine serum, p.s. 2%, and 2% Glu were placed in a water bath at 37°C set aside. For the preparation of the underlying agarose in a 6-well plate, the quantity for a hole, for example, about 1 mL of the mixture, and double quantity of DMEM culture were taken into the sterile centrifuge tube to make this volume containing 1.2% agarose then were mixed evenly and gently to avoid air bubbles. The mixture was added to the cell culture plate and was placed in the incubator at 37°C and 5% CO_2_. After solidification at room temperature, 1 × 10^3^ cells were taken into the 0.5 mL of the mixture in DMEM medium, added volume in 0.7% agarose, mixed evenly, and spread on the bottom glue. It was made to form double-layer agarose; each group had 3 wells. In order to avoid scald cells, agarose temperature should not be more than 40°C. After double-layer agarose preparation, cell culture plates were kept in the incubator for two weeks at 37°C and 5% CO_2_. The formation of cell clones was observed under the inverted light microscope.

### 2.5. Tumorigenicity Analysis in SCID Mice

Tumor-positive cell suspension was made with the cell concentration of 1 × 10^6^/mL. SCID mice (females, 6 weeks) were purchased from Beijing HFK Bioscience Co. Ltd. They were divided into two groups in random; each group consisted of three mice. Each group of mice was given an injection of 0.2 mL cell suspension, respectively, in the forelimb subcutaneously. Tumor growth and size in mice were observed regularly. After six weeks, tumor size was measured and tumor tissues were fixed with formalin for pathological sectioning. All experiments were performed according to the protocol approved by ethical committee of Beijing University of Technology and according to recommended guidelines for the care and use of laboratory animals.

### 2.6. Detecting 3-MC in Water Samples with the HPLC-MS Method

3-Methylcholanthrene (3-MC/MCA) is an important carcinogen and is often used by researchers to induce tumors in rodents. 3-MC in water samples was detected by high-performance liquid chromatography-tandem mass spectrometry (HPLC-MS/MS). This analysis was carried out on a Thermo TSQ Quantum Discovery Max triple quadrupole tandem mass spectrometer interfaced with a Thermo Finnigan HPLC system (Thermo Finnigan, San Jose, CA). The atmospheric pressure chemical ionization (APCI) was performed in the positive mode. The fractions of 3-methylcholanthrene (3-MC) were separated with a 2.1 mm × 150 mm (5 *μ*m in particle size) Zorbax SB-C18 column (Agilent Technologies, Palo Alto, CA) and eluted at a flow rate of 0.2 mL/min. The injection volume was 10 *μ*L. The mobile phase consisted of deionized 2 mM ammonium acetate with 0.1% acetic acid (solvent A) and acetonitrile (solvent B). An isocratic elution was conducted with 70% A and 30% B for 45 min. The instrumental parameters of the mass spectrometer were set as follows: vaporizer temperature 400°C; discharge current 3 *μ*A; ion transfer capillary temperature 250°C; source CID offset 15 V; sheath gas (nitrogen) pressure 30 psi; and aux gas (nitrogen) pressure 5 psi. The collision energy was set to 25 eV using argon at 1.0 mTorr. The analysis of 3-MC was performed by selecting reaction monitoring (SRM) with the transition of *m*/*z *269[M + H]^+^ → 254[M + H − CH_3_]^+^.

### 2.7. Statistical Analysis

Statistical analysis was performed by using GraphPad Prism. The results were expressed as means ± standard error of the mean (SEM). Comparisons between groups were assessed by *t*-test. *P* values of <0.05 was considered to indicate statistically significant differences.

## 3. Results

### 3.1. Detection of HPV DNA in Tissue Samples of Esophageal Cancer Patients

Statistical analysis was done after HPV DNA detection test in esophageal cancer tissue samples (Shenqiu People's Hospital). Test results showed that HPV (L1), HPV16, HPV18, HPV16 + 18 mix, HPV16 + 18-positive rate is quite high (L1: 68.2%, HPV16: 51.5%, and HPV18: 50%); other types were also detected. The details are shown in Table 3.

### 3.2. Tumorigenicity Analysis of Water Samples in Cell Transformation Assay

**Figure 1:**
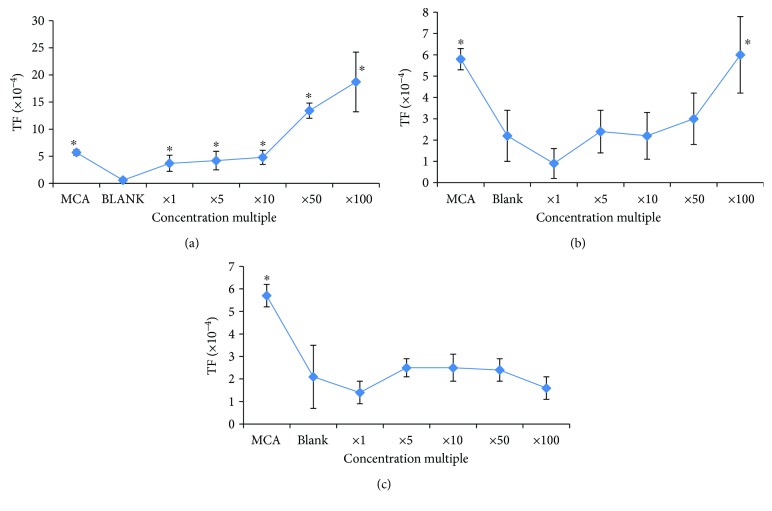
Cell transformation frequency of Balb/c 3T3 cells with different concentration multiples of different water samples. (a) River water sample; (b) well water sample from 10-meter depth; (c) well water sample from 40-meter depth. ^∗^*P* < 0.05.

A water sample from the river, a well water sample from 10-meter depth, and a well water sample from 40-meter depth were the three kinds of water sources used in cell transformation experiments for tumorigenicity. Methylcholanthrene (MCA) was used as a positive control. A river water sample and a shallow well water sample from 10-meter depth in the Balb/c 3T3 was showing high transformation frequency (TF) while a deep well water sample from 40-meter depth was showing significantly different result. The transformation frequency is dose dependent (Figure 1). In river water, TF is higher than MCA showing that many other contributing factors are present there.

**Figure 2:**
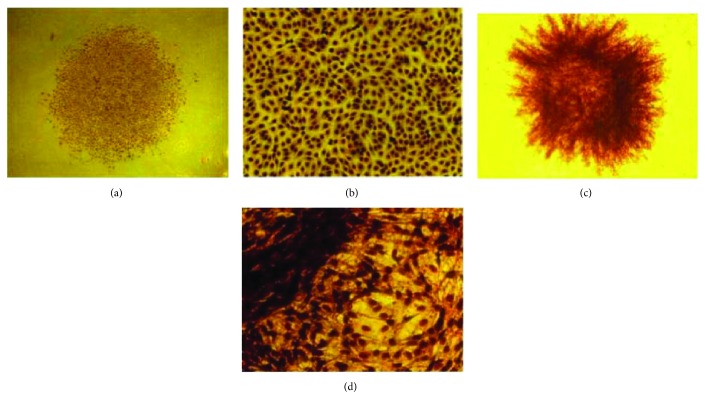
The clonal foci formed by normal Balb/c 3T3 cells (a, b) and transformed foci after malignant transformation (c, d) (Giemsa staining).

At the same time, there were significant morphological differences found after cell transformation when the cells were observed by Giemsa staining. Unconverted normal Balb/c 3T3 cells maintained in contact inhibition characteristics, clonal cells form foci remain in close contact, and monolayer growth cells were spindle-shaped with shallow nuclear staining (Figures 2(a) and 2(b)). The malignant transformed cells showed deep staining of the nucleus. The cells were multilayered and showed invasive growth. The uneven edges of the transformation foci invaded the normal single layer (Figures 2(c) and 2(d)).

### 3.3. Tumorigenicity Analysis of Water Samples in SCID Mice

**Figure 3:**
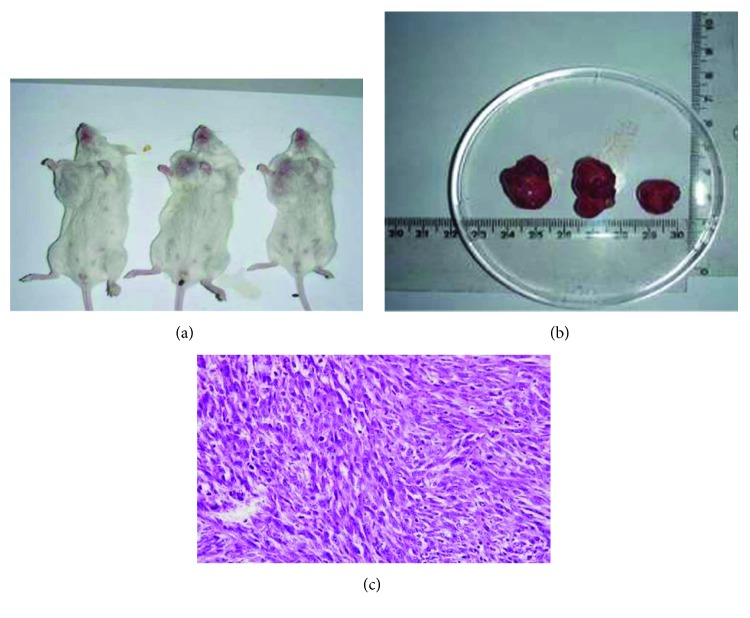
Tumorigenesis of malignant transformation of Balb/c 3T3 cells in SCID mice. (a) SCID mouse group injected with transformed Balb/c 3T3 cells. (b) Tumor growth in SCID mice. (c) Pathological section of tumor tissue stained with hematoxylin-eosin.

Nontransformed and transformed Balb/c 3T3 cells were inoculated into separate groups of SCID mice. The group with transformed cells forms tumors, and the tumor formation rate was 100% within 14 days. After 6 weeks of inoculation, tumor diameter was 13 ± 3 mm. Tumors, skin adhesion, and ulceration display a high degree of malignancy. Pathology showed a high degree of malignant fibrosarcoma tumor ulceration and local infiltration of inflammatory cells (Figures 3(a)–3(c)). Group inoculated with nontransformed cells showed no tumor formation.

### 3.4. Detection of 3-MC in Drinking Water Samples

**Figure 4:**
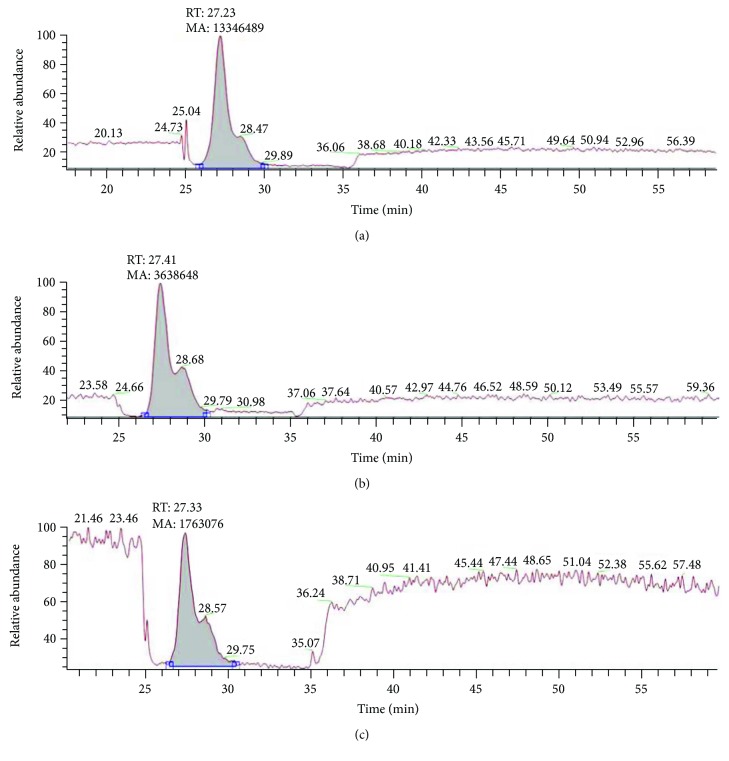
HPLC-MS of 3-MC in Huaihe River Basin water samples. (a) River water; (b) well water from 10-meter depth; and (c) well water from 40-meter depth.

For the detection of 3-MC from Huaihe River Basin water samples, HPLC-MS/MS was done. It can be seen that three water samples from the Shenqiu County of Henan province (collected from different depths) contain 3-MC. It was found that the concentration of 3-MC was higher in the river water sample than the water sample taken from 40-meter depth, while the concentration of 3-MC was higher in the well water sample taken from 10-meter depth as compared to the water sample taken from 40-meter depth (Figure 4).

## 4. Discussion

Air and water pollutions are the main environmental risk factors and major sources of morbidity and mortality in China. Hundreds of millions of people have come out of poverty during the past 15 years in China due to the rapid increase in economic growth and many-fold increase in gross domestic production [12, 23]. Now, the main concerns to the citizens of China and policymakers are the environmental effects which are produced as a result of this rapid economic growth. So, policymakers are facing the challenges of environmental pollution which is affecting human health badly. Despite poor air and water quality, half of China's water resources are considered too polluted for human use. It is estimated that there are 2.4 million premature deaths every year from different diseases like cardiopulmonary and gastrointestinal diseases and cancer because of different environmental risk factors [23–26].

Huaihe River flows through five provinces: Henan, Hubei, Anhui, Shandong, and Jiangsu, including 189 counties (including Shenqiu County). The basin area is 270,000 km^2^. The total population is about 165 million [27]. Since the 1970s, there is rapid development in social and economic areas in the Huaihe River Basin. Along with this development, this area is also facing a new water-related disaster which is water pollution. Because of discharge of accumulated wastewater, the mainstream of the river is polluted and caused the deterioration of drinking water quality in Shenqiu County as well as created the shortage of drinking water for the hundred thousand people living there. About 200 large water pollution incidents had occurred only in the Huaihe River, by the end of 1998 [6].

There are highly developed agriculture and intensive land cultivation with a large population in the Huaihe River Basin. Every year, the capacity of the reservoirs is decreasing and there is also deterioration of water quality as sediment and contaminants are entering the water system due to soil erosion and serious nutrient loss. A lot of data is available showing the harmful effects of pollution on human health but the research from Shenqiu County is not showing enough data. Environmental pollution in Huaihe River and the local cancer high-risk problem have appeared in recent years [6]. Areas having low cancer rates in the past now have high digestive tract cancer rates due to changes in the environment. The analysis of our experimental results showed that surface water has more pollutants and contaminants as compared to water from different depths. Surface water showed significant infiltration as it is documented in different published articles which provide the bases to speculate the causes of the Huaihe River pollution: (1) the absence of centralized water supply system, (2) the rural life wastewater directly thrown into the water bodies, and (3) the infiltrated residue of pesticides and factory pollution. Pollution was positively linked to high cancer incidents in a number of cancer village across Henan province, Jiangsu province, and Anhui province by Chinese Center for Disease Control and Prevention [6].

The strength of the toxicity is as follows: river water sample > well water sample from a 10-meter depth > well water sample from a 40-meter depth. For malignant cell transformation, no carcinogenicity is found in the well water sample from a 40-meter depth, while the well water sample from a 10-meter depth is found to have less carcinogenicity than the river water sample. The river water as well as shallow water is polluted and can induce cell malignancy and cause cancer risk in rural areas. Deep well water samples taken from a 40-meter depth are not contaminated, and there is no clearly induced cell malignancy and risk of cancer. We found no obvious toxicity in well water from a 40 m depth. Mutagenic activity was also found in drinking water of different U.S. cities with dose-dependent mutagenesis by residual organics [28]. The Huaihe River water is obviously carcinogenic and can induce cell malignant transformation and carcinoma. Water pollution was positively linked with EC as a risk factor [29–31]. This shows that as the depth of water increases, there is a trend of gradual decrease in pollution.

By testing of the genomic DNA of EC patients residing in the Huaihe River Basin in Shenqiu County, the HPV-positive rate is found very high which explains that the occurrence of EC may have some close relationship. A positive correlation is found with EC in different studies [32–34].

Water pollution and HPV infections may be the main causes for the high incidence of digestive tract cancer in Huaihe River Basin; hence, their synergistic effect plays a very important role in carcinogenesis. Therefore, in order to explore further the synergistic effect of water pollution and HPV infection for causing cancer, this study provides some basic but important information.

In conclusion, deep well water is good but pollutants are found in surface water, and shallow water resources are also partially polluted due to infiltration of pollutants. This can be one cause of local cancers. The pervasive presence of high-risk type HPV can be another cause of cancer. The prevalence of HPV and pollution has the synergistic effect and may be responsible for the high incidence of local tumors. This conclusion provides an important reference for comprehensive prevention and treatment of the local upper gastrointestinal tumor.

## Figures and Tables

**Table 1:** The general primer sets for HPV DNA detection.

Primer name	Sequences
GP5+	5′-TTTGTTACTGTGGTAGATACTAC-3′
GP6+	5′-GAAAAATAAACTGTAAATCATATTC-3′
My09	5′-CGTCCMARRGGAWACTGATC-3′
My11	5′-GCMCAGGGWCATAAYAATGG-3′
HPV16F	5′-CAACAAGACATACATCGACCG-3′
HPV16R	5′-TGGAACAACATTAGAACAGCAA-3′
HPV18F	5′-GCGCTTTGAGGATCCAACAC-3′
HPV18R	5′-ATTCAACGGTTTCTGGCAC-3′

21 HPV GenoArray diagnostic kit (Hybribio Limited©) was also used for rapid and accurate HPV genotyping. It can identify 21 human papillomaviruses including 15 high-risk HPV types (HPV16, 18, 31, 33, 35, 39, 45, 51, 52, 53, 56, 58, 59, 66, and 68) and 6 low-risk HPV types.

**Table 2:** The information of the sample point in the Huaihe River Basin of Shenqiu County, Henan province.

Sample type	Water volume (L)	Constant volume (mL)	Collected samples
River water	10	0.5	A tributary of the river
Shallow well water	10	0.3	About 10-meter depth
Deep well water	10	0.5	About 40-meter depth

**Table 3:** HPV detection in esophageal cancer tissue samples.

Group	Number of samples	Positive (%)	Negative (%)
HPV (L1)	66	45 (68.2)	21 (31.8)
HPV16	66	34 (51.5)	32 (48.5)
HPV18	66	33 (50.0)	33 (50.0)
HPV16 + 18 mix	66	26 (39.4)	40 (60.6)
HPV16/18	66	41 (62.1)	25 (37.9)
HPV51	66	3 (4.5)	63 (95.5)
HPV33	66	2 (3.0)	64 (97.0)
HPV52	66	1 (1.5)	65 (98.5)
HPV53	66	1 (1.5)	65 (98.5)
HPV58	66	1 (1.5)	65 (98.5)
HPV45	66	1 (1.5)	65 (98.5)
